# Correlations between obstructive sleep apnea and adenotonsillar hypertrophy in children of different weight status

**DOI:** 10.1038/s41598-019-47596-5

**Published:** 2019-08-07

**Authors:** Jing Wang, Yu Zhao, Wen Yang, Tian Shen, Pei Xue, Xiaohong Yan, Danni Chen, Yixin Qiao, Min Chen, Rong Ren, Jianjun Ren, Yang Xu, Yongbo Zheng, Jian Zou, Xiangdong Tang

**Affiliations:** 1 Department of Oto-Rhino-Laryngology, West China Hospital, West China Medical School, Sichuan University, Chengdu, Sichuan China; 2 Sleep Medicine Center, West China Hospital, Sichuan University, Chengdu, Sichuan China

**Keywords:** Paediatric research, Risk factors, Respiratory tract diseases

## Abstract

The present study aimed to evaluate the relationship between OSA and adenotonsillar size in children of different weight status. A total of 451 patients aged 2–13 years with suspected OSA were retrospectively enrolled in the study. Correlations between the apnea-hypopnea index (AHI) and adenotonsillar size in different weight status were investigated. The adenoidal/nasopharyngeal (A/N) ratio of underweight children was significantly higher than that of normal-weight children (*P* = 0.027). Both adenoid and tonsil size were positively correlated with logAHI in children of normal weight (r = 0.210, *P* = 0.001; and r = 0.212, *P* = 0.001) but uncorrelated in the other groups. Gender (OR = 1.49, 95% CI: 1.01–2.20, *P* = 0.043), obese (OR = 1.93, 95% CI: 1.10–3.40, *P* = 0.012), A/N ratio (OR = 1.55, 95% CI: 1.28–1.88, *P* < 0.001) and tonsil size (OR = 1.36, 95% CI: 1.18–1.57, *P* < 0.001) were all associated with the severity of OSA. Adenotonsillar hypertrophy contributed to OSA in normal-weight children. In children of abnormal weight, instead of treatment for adenotonsillar hypertrophy, appropriate treatments for other factors are required.

## Introduction

Sleep-disordered breathing (SDB) is highly prevalent in the general population ranging from primary snoring to obstructive sleep apnea (OSA)^1^. The OSA prevalence in children is 1–3%^2–4^, characterized by partial or complete obstruction of the upper airway during sleep, is associated with pulmonary hypertension, ventricular hypertrophy^5,6^, behavioral and cognitive problems such as impulsivity, anxiety, aggression and hyperactivity^7^. Polysomnography (PSG) is recommended as the gold standard for diagnosing OSA^8^. Adenotonsillar hypertrophy and craniofacial anomalies have been demonstrated as factors that increase the incidence of OSA^9,10^. Besides anatomic factors, obesity has been also suggested as contributor to OSA. Sakamoto *et al*.^11^ observed that SDB is more prevalent in obese than in non-obese children. In addition, Kang *et al*.^12^ found that obesity increased OSA risk in children. Furthermore, one study indicated underweight may also contribute to OSA. However, the pathophysiology of remains unclear^13^. Dayyat *et al*.^14^ indicated that non-obese children may have larger adenotonsillar than obese children. The magnitude of adenotonsillar hypertrophy may be smaller in obese children. However, these studies didn’t analyse the correlation between different degree of adenotonsillar size with the severity of OSA in underweight, normal-weight, overweight, and obese children, respectively. Adenotonsillectomy and medical therapies are both commonly used to treat pediatric OSA patients with adenotonsillar hypertrophy. Obese children may be at greater risk of residual disease after adenotonsillectomy^15,16^. Medical therapies, such as nasal corticosteroids or montelukast, are minimally effective in obese children^17^. We hypothesized that adenotonsillar hypertrophy may not be the major contributor to OSA severity in obese children. Meanwhile, adenotonsillectomy has only a modest effect in OSA children with craniofacial abnormalities or neuromuscular disorders^18–20^. Therefore, it is important to define risk factors for OSA that may allow treatment individualization.

To date, the relationship between adenotonsillar size and OSA severity in children of different weight status has not been defined. Here, we divided children into four groups by weight status. Polysomnography was used to evaluate OSA severity. The effects of age, gender, and adenoid and tonsil sizes on OSA severity were explored. The results yielded indications for prescription of operative and medical therapies for children with OSA exhibiting adenotonsillar hypertrophy.

## Methods

### Study design and participants

This cross-sectional study was approved by the Ethics Committee of West China Hospital (approval #146, 2018) and was registered at chictr.org (ChiCTR1800017895). The study was performed in accordance with relevant guidelines and regulations. Written informed consent was obtained from all of the parents. We included children who were diagnosed at the Department of Otolaryngology of West China Hospital in Chengdu, China from January 2013 to June 2018. Patients aged 2–13 years were eligible for inclusion if they were suspected to have OSA, underwent full-night polysomnography (PSG), and complete clinical data were available. Patients with any craniofacial abnormality, any genetic or neuromuscular disease, or who had undergone adenotonsillectomy were excluded. OSA was defined as an apnea-hypopnea index (AHI) ≥1 event/h. The primary snoring (control) group included children with AHI <1 event/h.

### Clinical data

We collected polysomnographic data and electronic medical records; we recorded age, gender, height, weight, and tonsil and adenoid sizes.

#### Tonsil size

Tonsils were graded using the Brodsky scheme^21^. The tonsil size was the sum of the sizes of the two sides of either tonsil; this reduces any effect of tonsil asymmetry. The grades were defined as follows: grade I, small tonsils confined to the tonsillar pillars; grade II, tonsils extending just outside the pillars; grade III, tonsils extending outside the pillars, but not meeting in the midline; and grade IV, large tonsils meeting at the midline.

#### Adenoid size

**Figure 1:**
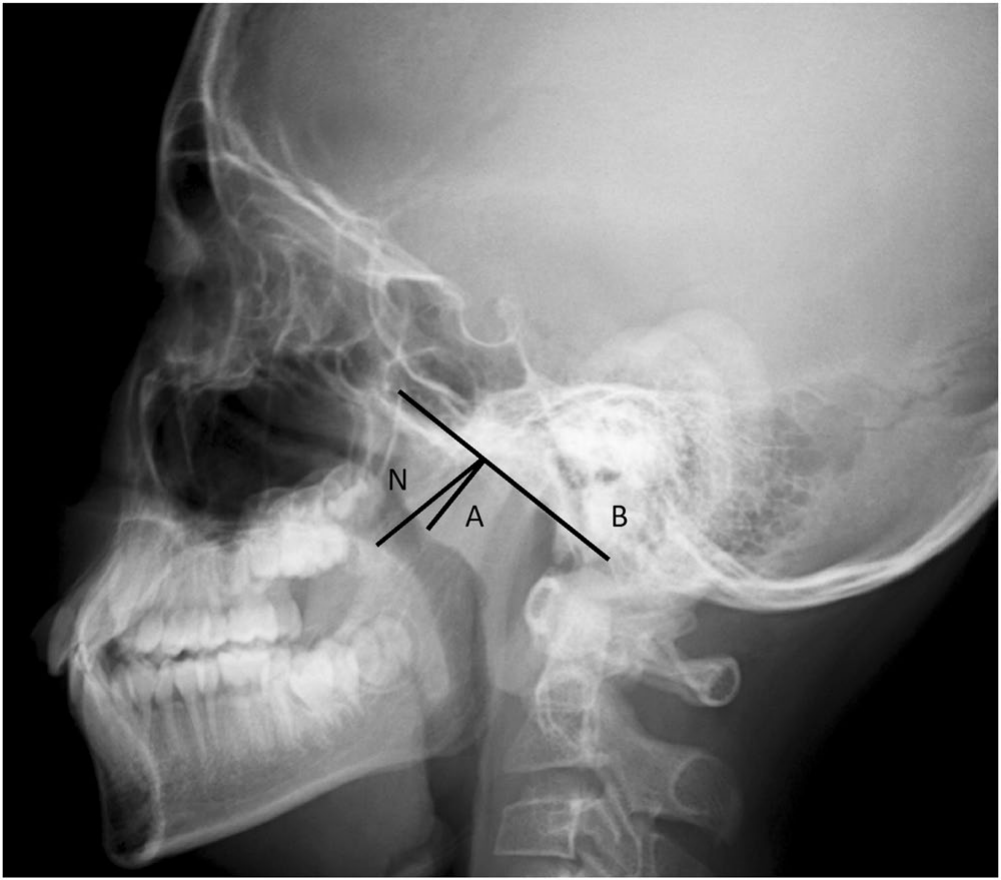
Measurement of the adenoidal/nasopharyngeal (A/N) ratio on lateral cephalometric radiographs. The distance A is measured along the line running perpendicular from the point of maximal convexity along the inferior margin of the adenoidal shadow to the point of intersection with line B. The nasopharyngeal space N is the distance between the posterior/superior edge of the hard palate and the anterior/inferior edge of the sphenobasioccipital synchondrosis. The A/N ratio is obtained by dividing A by N.

Adenoids were measured using the method of Fujioka^22^. We measured the adenoidal/nasopharyngeal (A/N) ratio on lateral cephalometric radiographs and then calculated adenoid sizes. The A parameter is the distance from the point of maximal convexity along the inferior margin of the adenoidal shadow to the line B drawn along the straight region of the anterior basiocciput margin. A is measured along the line drawn perpendicular from the point of maximal convexity along the inferior margin of the adenoidal shadow to its point of intersection with line B. The nasopharyngeal parameter N is the distance between the posterior/superior edge of the hard palate and the anterior/inferior edge of the sphenobasioccipital synchondrosis. The A/N ratio is obtained by dividing A by N (Fig. 1). An A/N ratio >0.67 was considered to indicate adenoidal hypertrophy.

#### Weight status

We divided children into four groups by age- and gender-corrected body mass index (BMI) using the recognized Chinese guidelines^23^. The underweight group included those of BMI ≤ percentile 5; the normal-weight group, children of BMI > percentile 5 but < percentile 85; the overweight group, children of BMI > percentile 85 but < percentile 95; and the obese group, children of BMI > percentile 95.

#### Polysomnography

All patients underwent full-night polysomnography (Philips Respironics, Bend, OR, USA). In line with the criteria of the American Academy of Sleep Medicine. Apnea was defined as a >90% reduction in airflow for at least the duration of 2 breaths, hypopnea as a ≥30% reduction of airflow for at least 2 breaths associated with a ≥3% desaturation from pre-event baseline or the event is associated with an arousal. The AHI was defined as the average number of apneas and hypopneas per hour of sleep. Mild disease was defined as an AHI ≥1 but <5 events/h; moderate disease by an AHI ≥5 but <10 events/h; and severe disease by an AHI ≥10 events/h^24^.

### Statistical analysis

SPSS software (ver. 22; IBM SPSS, Armonk, NY, USA) was used to perform all statistical analyses. Continuous data are expressed as means with standard deviations, and categorical variables as frequencies with percentages. Data that were normally distributed and that exhibited variance homogeneity were subjected to one-way analysis of variance with the Bonferroni post-hoc test, and other data were analyzed using the Kruskal–Wallis test. Categorical variables were compared using the chi-squared test. Correlations between the AHI and adenotonsillar size were investigated with the aid of the Pearson test and Spearman test. We used multivariate logistic regression to seek independent predictors of OSA. A P-value < 0.05 was considered to reflect statistical significance.

## Results

### Patient characteristics

We included 451 children of mean age 5.5 ± 2.4 years (69.6% boys), who were divided into underweight, normal-weight, overweight, and obese groups. We enrolled more males than females, but there was no significant difference of sex ratio among the four groups. The baseline demographics and PSG parameters of the four groups were summarized in Table 1. Compared to the underweight (*P* = 0.034), normal-weight (*P* < 0.001) and overweight group (*P* = 0.018), obese children had a significantly higher AHI. Both apnea index (*P* = 0.037) and obstructive AHI (*P* = 0.022) were significantly higher in obese children than those in normal weight children. In addition, obese children had a significantly higher hypopnea index and desaturation index than those in underweight (*P* = 0.007 and *P* = 0.001), normal-weight (*P* < 0.001 and *P* < 0.001), and overweight children (*P* = 0.002 and *P* = 0.002). The A/N ratio of underweight children was significantly higher than that of normal-weight children (*P* = 0.027). However, tonsil size did not differ among the four groups.

**Table 1:** Demographic and clinical characteristics by weight status.

Variable	Underweight (n = 62)	Normal-weight (n = 273)	Overweight (n = 52)	Obese (n = 64)	*P*-value
Age, years	5.16 ± 1.77	5.56 ± 2.40	5.81 ± 2.74	5.69 ± 2.45	0.476
Gender, male (No. %)	42 (67.74)	186 (68.13)	33 (63.46)	54 (84.38)	0.063
BMI, kg/m^2^	12.94 ± 0.91^d^	15.58 ± 1.21^c^	18.41 ± 1.85^b^	20.94 ± 2.56^a^	<0.001
A/N ratio	0.69 ± 0.11^a^	0.65 ± 0.10^b^	0.67 ± 0.08^a,b^	0.66 ± 0.09^a,b^	0.034
Tonsil size	6.52 ± 1.41	6.66 ± 1.26	6.79 ± 1.11	6.83 ± 1.28	0.498
TST, min	492.27 ± 53.67	477.86 ± 52.25	464.71 ± 59.99	473.69 ± 71.65	0.067
Sleep efficiency, %	90.97 ± 6.87	89.54 ± 7.96	88.01 ± 8.26	87.41 ± 9.97	0.059
N1, %	12.91 ± 6.45	14.94 ± 9.70	14.10 ± 7.67	14.98 ± 8.65	0.411
N2, %	40.00 ± 10.22	40.25 ± 10.50	40.41 ± 9.26	40.99 ± 9.49	0.949
N3, %	29.47 ± 8.54	26.79 ± 8.02	29.26 ± 7.33	27.33 ± 8.35	0.040
REM, %	17.63 ± 5.28	17.90 ± 5.60	16.23 ± 4.67	16.71 ± 4.32	0.111
Awakenings	12.60 ± 8.68	13.92 ± 9.86	14.35 ± 9.79	14.28 ± 8.68	0.716
Total arousal index, h TST	11.99 ± 8.92	11.63 ± 5.81	12.34 ± 6.80	13.30 ± 9.81	0.392
Respiratory arousal index, h TST	3.51 ± 5.29	2.85 ± 3.90	3.86 ± 5.21	4.44 ± 6.24	0.047
AHI	17.03 ± 18.51^b^	13.53 ± 14.90^b^	15.94 ± 18.65^b^	25.75 ± 25.18^a^	<0.001
Apnea index, h TST	7.09 ± 12.60^a,b^	5.38 ± 9.11^b^	7.00 ± 12.06^a,b^	9.42 ± 12.61^a^	0.043
Hypopnea index, h TST	9.94 ± 10.92^b^	8.15 ± 8.82^b^	8.94 ± 10.02^b^	16.34 ± 17.82^a^	<0.001
Obstructive AHI, h TST	5.69 ± 10.56^a,b^	4.21 ± 8.45^b^	5.89 ± 11.61^a,b^	8.13 ± 11.82^a^	0.029
Minimal SaO_2_, %	73.23 ± 14.25	74.84 ± 12.96	76.15 ± 13.26	71.19 ± 14.68	0.155
Mean oxygen saturation, %	94.82 ± 4.08	95.73 ± 1.71	95.38 ± 2.90	95.08 ± 2.88	0.031
Desaturation index, h TST	17.90 ± 19.38^b^	14.92 ± 15.32^b^	18.01 ± 18.65^b^	30.41 ± 28.06^a^	<0.001

The values are means ± standard deviations. Gender is expressed as frequencies with percentages. A/N ratio, adenoidal/nasopharyngeal ratio; TST, total sleep time; N, no rapid eye movement; REM, rapid eye movement; AHI, apnea-hypopnea index. Means with different lower case letters represent a statistically significant difference within each different groups (*P* < 0.05).

### Correlation between adenotonsillar size and the logAHI

**Figure 2:**
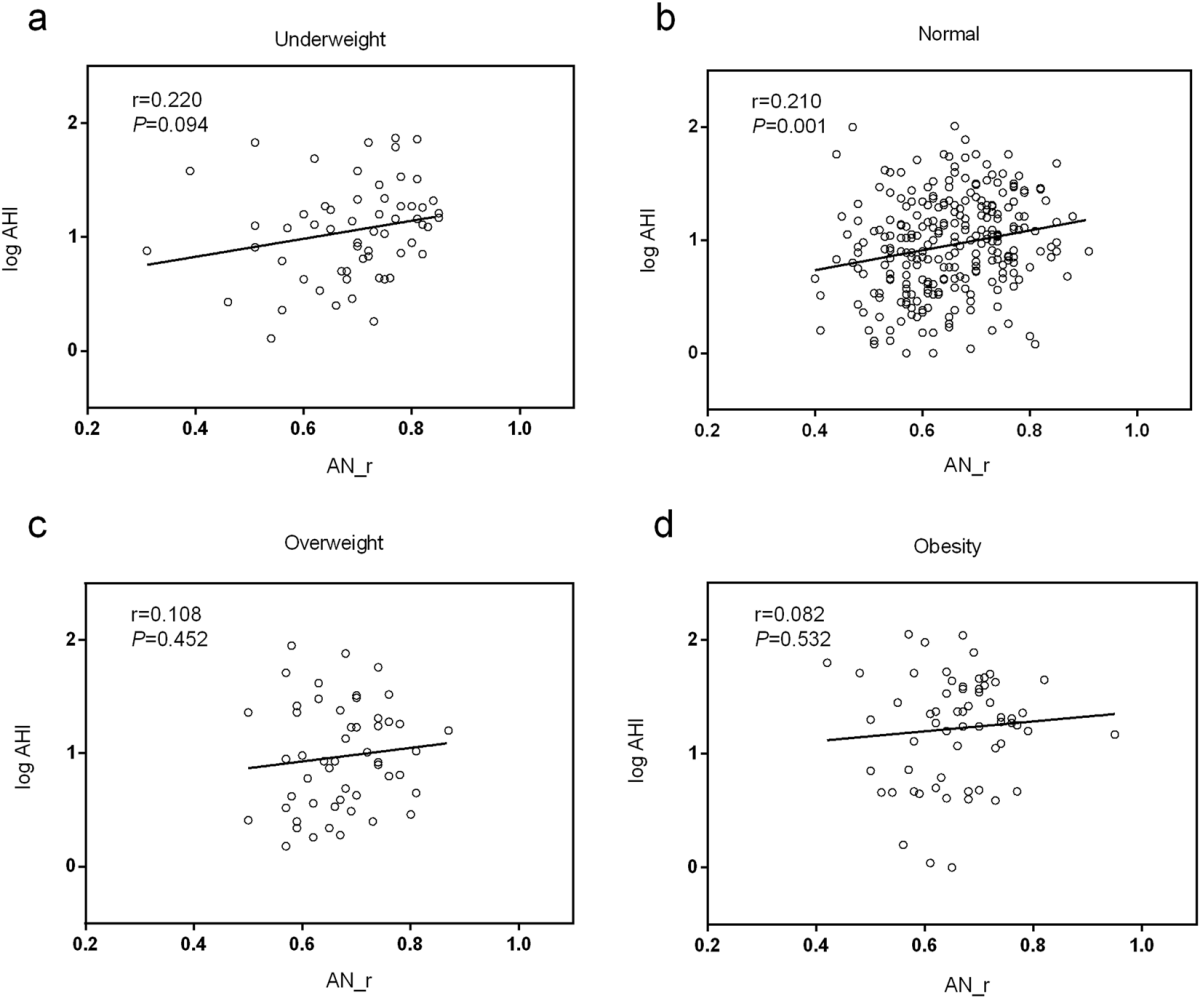
Relationships between adenoidal size and logAHI in children of different weight status (**a**,**c**,**d**). No correlation between adenoidal size and logAHI was evident in the underweight, overweight, or obese groups. (**b**) Adenoidal size was positively correlated with logAHI in the normal-weight group (r = 0.210; *P* = 0.001).

**Figure 3:**
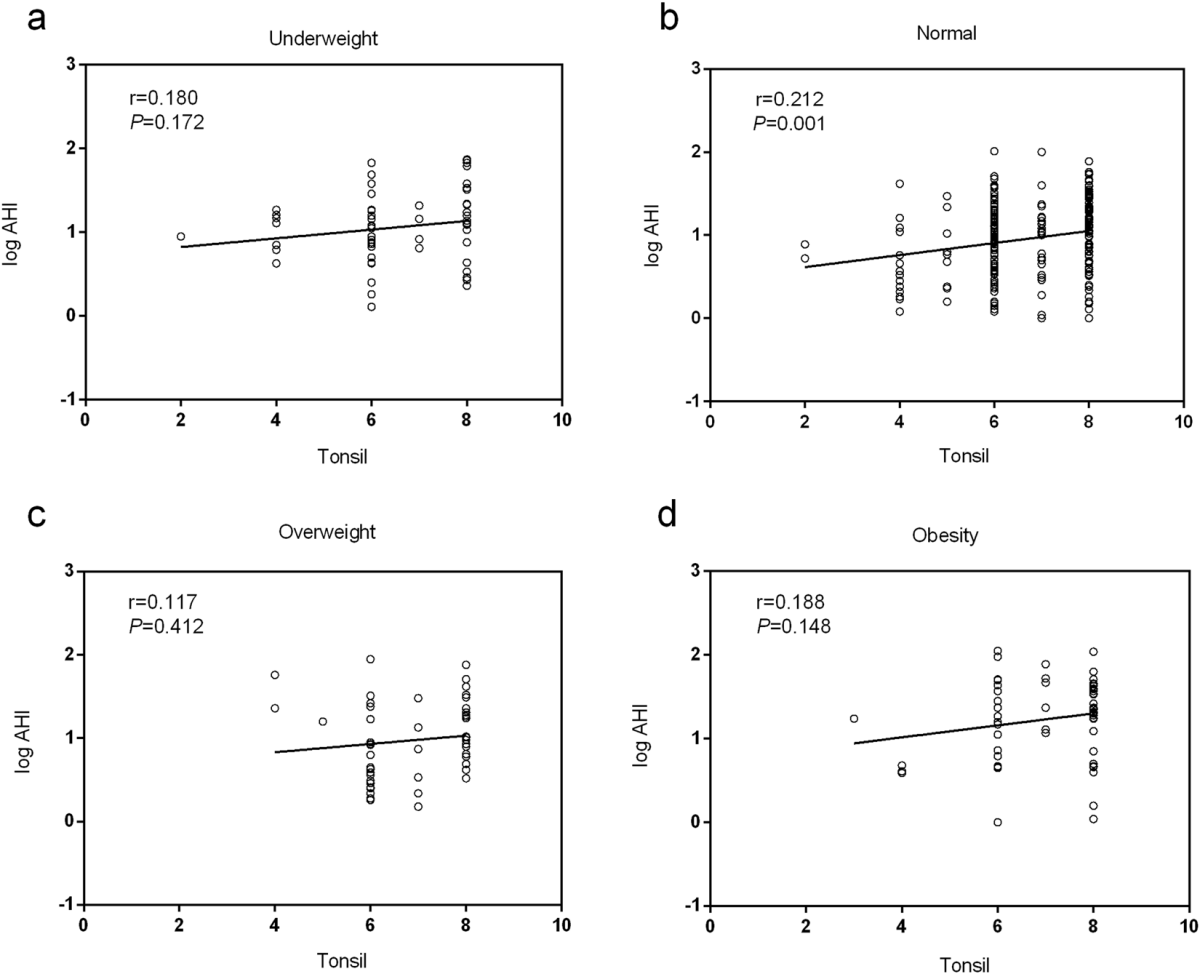
Relationships between tonsil size and logAHI in children of different weight status (**a**,**c**,**d**). No correlation between tonsil size and logAHI was evident in the underweight, overweight, or obese groups. (**b**) Tonsil size was positively correlated with logAHI in the normal-weight group (r = 0.212; *P* = 0.001).

Figures 2 and 3 presents how differrent adenotonsillar size and the severity of OSA are related in. As shown in Fig. 2, adenoid size was positively associated with the logAHI in the normal-weight group (r = 0.210; *P* = 0.001) but not in the underweight, overweight or obese groups. Figure 3 shows that tonsil size was positively correlated with the logAHI in the normal-weight group (r = 0.212; *P* = 0.001) but uncorrelated in the other groups.

### Multivariable logistic regression

**Figure 4:**
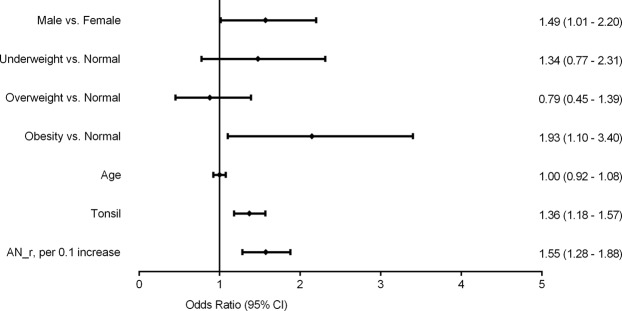
Forest plot of data from multivariate logistic regression revealing factors independently associated with the severity of pediatric OSA. CI = confidence interval.

We used multivariable ordinal logistic regression to define risk factors for OSA in children. We found gender (odds ratio: 1.49, 95% CI: 1.01–2.20, *P* = 0.043), the A/N ratio (odds ratio: 1.55, 95% CI: 1.28–1.88, *P* < 0.001) and tonsil size (odds ratio: 1.36, 95% CI: 1.18–1.57, *P* < 0.001) independently predicted OSA severity after adjustment for age. Obese (compared to normal-weight) children were at higher risk of more severe OSA (odds ratio: 1.93, 95% CI: 1.10–3.40, *P* = 0.012) (Fig. 4).

## Discussion

Mental spine - clivus length and intermandibular length of children increase linearly with the age. Meanwhile, tonsils and adenoid grow proportionally to the skeletal structures at the same time^25^ A longitudinal observational study showed that the tonsil and adenoid grow to maximum size at about ages of 7–9 and of 12–13, respectively^15,26^ In this study, we included the children aged from 2 to 13 years old, who were the most common candidates for adenotonsillectomy. The results of the present study may be used to evaluate the surgical indications in children with OSA.

According to previous studies, obesity is a risk factor for OSA^27,28^, which is consistent with our results. Some studies have suggested that OSA is a disease of inflammation^29^. Intermittent airway obstruction places mechanical stress on mucosa that promotes local airway inflammation^30^. As obesity is also a systemic inflammatory disease, it may exacerbate this process and contribute to OSA. Furthermore, fat deposition at specific sites may also contribute to the development of OSA, which can lead to increased collapsibility of the upper airways, as well as decreased chest compliance and functional residual capacity^31^. In this study, we found that although obese children exhibited a significantly higher AHI than underweight, normal-weight and overweight children, the adenotonsillar size did not differ when obese children were compared with underweight, normal-weight, and overweight children. Adenoid and tonsil sizes were not associated with AHI in obese children. Our findings were similar to another study, which found less adenotonsillar hypertrophy in obese children at any given level of AHI compared to nonobese children^14^. Arens *et al*.^32^. Used magnetic resonance imaging to compare the airway structure and body fat composition between obese children with or without OSA. They found that BMI was not correlated to the size of lymphoid tissues. Lymphoid proliferation in OSA may be independent of obesity. Meanwhile, Several studies found obese children responded poorly to adenotonsillectomy^17^ and were more likely to exhibit residual disease^15,16^. The cause may be adenotonsillectomy can not eliminate the inflammation induced by obesity. Another cause may be fat deposition at specific sites can not be eliminated by adenotonsillectomy. Therefore, we speculated that there were more risk factors for obese children than normal weight children with OSA. Adenotonsillar hypertrophy may not be the prime cause of OSA in obese children. Weight loss has a high success rate in treating obese adolescents with SDB^33,34^. However, there was no evidence regarding the efficacy of weight loss for obese children with SDB. This might be due to the difficult to control on weight loss on children. In our study, the A/N ratio of underweight children was significantly higher than that of normal-weight children. However, underweight was not a risk factor for OSA. These results revealed that adenoid hypertrophy may be the major contributors to OSA in underweight children. Moreover, both adenoid and tonsil sizes were not associated with AHI in underweight and overweight children. Very few studies have evaluated underweight or overweight children with OSA. One study indicated that underweight may contribute to OSA when used the criterion of AHI of ≥5 events/h. On the contrary, underweight may not be a risk factor of OSA when used the criterion of AHI of ≥2 events/h in the same study^13^. The most widely used criterion (which we employed) is AHI ≥1 event/h in the presence of SDB symptoms^17^. The difference of the ages of included patients and the classification criteria in the two studies might explain the discrepancy. Pediatric OSA diagnostic criteria remains controversial.

So far as a large numbers of study showing, adenotonsillar hypertrophy may plays a major role in pediatric OSA^12^, similar to our findings that adenotonsillar size independently predicted OSA severity. However, a systematic review of association between tonsil size and OSAS severity found the correlation between tonsil size and OSAS severity controversial^35^. The reasons for the differences may due to the tonsil size is measured subjectively. It is possible to be more accurate by objective measurements, such as CT scan or magnetic resonance imaging. In our study, we found the effects of adenotonsillar hypertrophy varied among different populations. Adenoid and tonsil sizes were positively associated with the logAHI in normal-weight children, but not in those who were underweight, overweight, or obese. Therefore, adenoid hypertrophy may be the major contributors to OSA in normal-weight children. Current treatments for adenotonsillar hypertrophy may be useful in normal-weight children.

Earlier studies indicated that boys were more prone to OSA than girls^3,28^. Similarly, We enrolled more males than females, but there was no significant difference of sex ratio among the four groups. Boys had highest percentages in obese children. The results were consistent with the increasing trend of the prevalence in males with obesity over the past years^36,37^. According to our study, male gender was an independent predictor of OSA severity after adjustment for age. The pathophysiological mechanism may involve a predisposition to pharyngeal collapse, reflecting the longer length of vulnerable airway and larger soft palate of males^38,39^. However, several studies found that gender was not a risk factor for pediatric OSA. Future epidemiological studies should explore the association between gender and OSA.

Our study had certain limitations. First, the numbers in the underweight, overweight and obese groups were relatively small. But we did the adjustment of data. Second, owing to ethical considerations, almost all of the children subjected to polysomnography and lateral cephalometric radiography had SDB, and we thus lacked a normal control group. Third, considering about the other factors for OSA, further evaluation of upper airway structure and neuromuscular function in the context of the OSA pathophysiological mechanisms at play in children of various weights status are needed.

## Conclusions

Gender, obesity, adenoid and tonsillar size were all associated with pediatric OSA severity. Adenotonsillar hypertrophy was a major risk factor for OSA in normal-weight children. In children of abnormal weight, other risk factors may be in play, and need be defined to allow for appropriate treatment.

## References

[1] Frye, S. S. *et al*. Childhood obesity, weight loss and developmental trajectories predict the persistence and remission of childhood sleep-disordered breathing. *Pediatr Obes* (2018). 10.1111/ijpo.12461 PMC6424126 30256539

[2] Li AM (2010). Epidemiology of obstructive sleep apnoea syndrome in Chinese children: a two-phase community study. Thorax.

[3] Lumeng JC, Chervin RD (2008). Epidemiology of pediatric obstructive sleep apnea. Proc Am Thorac Soc.

[4] Brunetti L (2001). Prevalence of obstructive sleep apnea syndrome in a cohort of 1,207 children of southern Italy. Chest.

[5] Ingram DG, Singh AV, Ehsan Z, Birnbaum BF (2017). Obstructive Sleep Apnea and Pulmonary Hypertension in Children. Paediatr Respir Rev.

[6] Amin RS (2002). Left ventricular hypertrophy and abnormal ventricular geometry in children and adolescents with obstructive sleep apnea. Am J Respir Crit Care Med.

[7] Smith DL, Gozal D, Hunter SJ, Kheirandish-Gozal L (2017). Frequency of snoring, rather than apnea-hypopnea index, predicts both cognitive and behavioral problems in young children. Sleep Med.

[8] Roland PS (2011). Clinical practice guideline: Polysomnography for sleep-disordered breathing prior to tonsillectomy in children. Otolaryngol Head Neck Surg.

[9] Redline S (1999). Risk factors for sleep-disordered breathing in children. Associations with obesity, race, and respiratory problems. Am J Respir Crit Care Med.

[10] Tagaya M (2012). Relationship between adenoid size and severity of obstructive sleep apnea in preschool children. Int J Pediatr Otorhinolaryngol.

[11] Sakamoto, N. *et al*. Sleep Duration, Snoring Prevalence, Obesity, and Behavioral Problems in a Large Cohort of Primary School Students in Japan. *Sleep* **40** (2017). 10.1093/sleep/zsw082 28364432

[12] Kang KT, Chou CH, Weng WC, Lee PL, Hsu WC (2013). Associations between adenotonsillar hypertrophy, age, and obesity in children with obstructive sleep apnea. PLoS One.

[13] Kang KT, Lee PL, Weng WC, Hsu WC (2012). Body weight status and obstructive sleep apnea in children. Int J Obes (Lond).

[14] Dayyat E, Kheirandish-Gozal L, Sans Capdevila O, Maarafeya M, Gozal D (2009). Obstructive sleep apnea in children: relative contributions of body mass index and adenotonsillar hypertrophy. Chest.

[15] Bhattacharjee R (2010). Adenotonsillectomy outcomes in treatment of obstructive sleep apnea in children: a multicenter retrospective study. Am J Respir Crit Care Med.

[16] Amin R (2008). Growth velocity predicts recurrence of sleep-disordered breathing 1 year after adenotonsillectomy. Am J Respir Crit Care Med.

[17] Kaditis AG (2016). Obstructive sleep disordered breathing in 2- to 18-year-old children: diagnosis and management. Eur Respir J.

[18] Shete MM, Stocks RM, Sebelik ME, Schoumacher RA (2010). Effects of adeno-tonsillectomy on polysomnography patterns in Down syndrome children with obstructive sleep apnea: a comparative study with children without Down syndrome. Int J Pediatr Otorhinolaryngol.

[19] Amonoo-Kuofi K (2009). Adenotonsillectomy for sleep-disordered breathing in children with syndromic craniosynostosis. J Craniofac Surg.

[20] Kerschner JE, Lynch JB, Kleiner H, Flanary VA, Rice TB (2002). Uvulopalatopharyngoplasty with tonsillectomy and adenoidectomy as a treatment for obstructive sleep apnea in neurologically impaired children. Int J Pediatr Otorhinolaryngol.

[21] Brodsky L, Moore L, Stanievich JF (1987). A comparison of tonsillar size and oropharyngeal dimensions in children with obstructive adenotonsillar hypertrophy. Int J Pediatr Otorhinolaryngol.

[22] Fujioka M, Young LW, Girdany BR (1979). Radiographic evaluation of adenoidal size in children: adenoidal-nasopharyngeal ratio. AJR Am J Roentgenol.

[23] Li H, Ji CY, Zong XN, Zhang YQ (2009). Height and weight standardized growth charts for Chinese children and adolescents aged 0 to 18 years. Zhonghua Er Ke Za Zhi.

[24] Berry RB (2012). Rules for scoring respiratory events in sleep: update of the 2007 AASM Manual for the Scoring of Sleep and Associated Events. Deliberations of the Sleep Apnea Definitions Task Force of the American Academy of Sleep Medicine. J Clin Sleep Med.

[25] Arens R (2002). Linear dimensions of the upper airway structure during development: assessment by magnetic resonance imaging. Am J Respir Crit Care Med.

[26] Ishida T (2018). Patterns of adenoid and tonsil growth in Japanese children and adolescents: A longitudinal study. Sci Rep.

[27] Lam YY (2006). The correlation among obesity, apnea-hypopnea index, and tonsil size in children. Chest.

[28] Goodwin JL, Vasquez MM, Silva GE, Quan SF (2010). Incidence and remission of sleep-disordered breathing and related symptoms in 6- to 17-year old children–the Tucson Children’s Assessment of Sleep Apnea Study. J Pediatr.

[29] Bhattacharjee R, Kim J, Kheirandish-Gozal L, Gozal D (2011). Obesity and obstructive sleep apnea syndrome in children: a tale of inflammatory cascades. Pediatr Pulmonol.

[30] Almendros I (2008). Upper airway collapse and reopening induce inflammation in a sleep apnoea model. Eur Respir J.

[31] Romero-Corral A, Caples SM, Lopez-Jimenez F, Somers VK (2010). Interactions between obesity and obstructive sleep apnea: implications for treatment. Chest.

[32] Arens R (2011). Upper airway structure and body fat composition in obese children with obstructive sleep apnea syndrome. Am J Respir Crit Care Med.

[33] Verhulst SL, Franckx H, Van Gaal L, De Backer W, Desager K (2009). The effect of weight loss on sleep-disordered breathing in obese teenagers. Obesity (Silver Spring).

[34] Van Eyck A (2018). Clinical Predictors of Residual Sleep Apnea after Weight Loss Therapy in Obese Adolescents. J Pediatr.

[35] Nolan J, Brietzke SE (2011). Systematic review of pediatric tonsil size and polysomnogram-measured obstructive sleep apnea severity. Otolaryngol Head Neck Surg.

[36] Skinner AC, Perrin EM, Skelton JA (2016). Prevalence of obesity and severe obesity in US children, 1999–2014. Obesity (Silver Spring).

[37] Sun H, Ma Y, Han D, Pan CW, Xu Y (2014). Prevalence and trends in obesity among China’s children and adolescents, 1985–2010. PLoS One.

[38] Malhotra A (2002). The male predisposition to pharyngeal collapse: importance of airway length. Am J Respir Crit Care Med.

[39] Ronen O, Malhotra A, Pillar G (2007). Influence of gender and age on upper-airway length during development. Pediatrics.

